# Serum testosterone acts as a prognostic indicator in polycystic ovary syndrome‐associated kidney injury

**DOI:** 10.14814/phy2.14219

**Published:** 2019-08-25

**Authors:** Yali Song, Wenting Ye, Huiyun Ye, Tingting Xie, Weiwei Shen, Lili Zhou

**Affiliations:** ^1^ Center for Reproductive Medicine, Department of Obstetrics and Gynecology Nanfang Hospital, Southern Medical University Guangzhou China; ^2^ State Key Laboratory of Organ Failure Research, National Clinical Research Center of Kidney Disease, Division of Nephrology Nanfang Hospital Southern Medical University Guangzhou China; ^3^ Guangzhou Regenerative Medicine and Health Guangdong Laboratory Guangzhou China

**Keywords:** testosterone, polycystic ovary syndrome, kidney, tubular cell, fibrotic injury

## Abstract

Polycystic ovary syndrome (PCOS) is closely related with the onset and development of metabolic abnormalities. However, the correlation between PCOS and kidney injury has not been clarified, and the underlying mechanism remains unknown. Herein, we performed a prospective survey in 55 PCOS and 69 healthy participants. Furthermore, the correlation analyses between serum testosterone and renal functional manifestations of patients and healthy subjects, including urinary albumin to creatinine ratio (UACR), urinary κ‐light chains (KapU), urinary λ‐light chains (LamU), urinary α1‐microglobulin (α1‐MU), and urinary β2‐microglobulin (β2‐MU), were analyzed. Compared with that in normal subjects, the levels of serum testosterone and UACR were significantly higher in PCOS patients. Serum testosterone is significantly correlated with the disease severity of PCOS. Although urinary excretions of KapU, LamU, α1‐MU, and β2‐MU did not increase in PCOS patients, they had a significantly positive correlation with the extent of serum testosterone in PCOS patients. *IN vitro,* primary cultured human ovary granulosa cells (GCs) were isolated from the follicular fluid (FF) extracting from PCOS patients and controls. FF, especially which extracted from PCOS patients with a high expression of serum testosterone, significantly induced cell apoptosis and inflammation in human GCs. To examine the communication between PCOS and kidney injury, a human proximal tubular epithelial cell line (HKC‐8) was cultured and administered FF. Interestingly, FF from PCOS patients with a higher level of serum testosterone induced fibrotic lesions in HKC‐8 cells. These data suggest serum testosterone plays a critical role in PCOS and PCOS‐associated kidney injury. Serum testosterone may serve as a promising indicator for kidney fibrotic injury outcomes in PCOS patients.

## Introduction

Polycystic ovary syndrome (PCOS) is the most common type of endocrine disorders in women of reproductive age. Up to 20% in women of childbearing age worldwide are suffering PCOS (Teede et al., 2010; Li et al., 2013; Azziz et al., 2016; Lizneva et al., 2016; Cooney and Dokras, 2018). PCOS dramatically affects reproductive capabilities and often result in infertility in women. It is manifested by oligomenorrhea or amenorrhea, oligoovulation or anovulation, and excessive secretion of testosterone (Reis and Honorato‐Sampaio, 2018; Showell et al., 2018; Wang et al., 2018). For the high prevalence of infertility, PCOS patients often develop substantial personal distress (Peterson et al., 2014; Santoro et al., 2016). These would certainly impose higher financial burden on societal health care. Notably, in United States, the total cost for evaluating and providing medical treatments to PCOS patients is up to $4.36 billion, of which $533 million (12.2% of the total) is used for providing infertility care. Hence, to develop a prognostic indicator in PCOS is of great value to provide early prevention and treatment to PCOS patients and release the heavy burden to the society.

PCOS is characterized by abnormal ovarian function and infertility. However, PCOS is also a type of metabolic dysfunction with a systemic disorder. A large amount of reports show PCOS intimately links to the disease progression of obesity, diabetes mellitus (DM), dyslipidemia, hypertension, anxiety, depression, obstructive sleep apnea, endometrial cancer, fatty liver, and cardiovascular diseases (Jakubowicz et al., 2013; Barry et al., 2014; Yang et al., 2016; Cooney et al., 2017; Kahal et al., 2017; Behboudi‐Gandevani et al., 2018; Cooney and Dokras, 2018; Teede et al., 2018). Conversely, these metabolic abnormalities may increase the risks of pregnancy complications such as gestational diabetes mellitus (GDM), pregnancy‐induced hypertension and preeclampsia (Yu et al., 2016), suggesting the reciprocal activation between PCOS and metabolic disorders.

Chronic kidney disease (CKD) is a gradual loss of kidney function over months to years. Without early preventing and treatment, CKD could eventually progress into the disease state of uremia, an end‐stage kidney disease with significantly increased mortality (Nigam and Bush, 2019). However, due to lack of early diagnostic methods, CKD patients would inevitably be ignored in an early stage of disease. The morbidity of CKD is highly associated with metabolic abnormalities of obesity, DM, and cardiovascular diseases (Castro and Coresh, 2009; Chadban et al., 2010; Piccoli et al., 2018). Notably, all of them are also common concomitant complications in PCOS, suggesting the high correlation between PCOS and kidney injury. Studies have shown a potential relationship between PCOS and kidney diseases. Over 50% of PCOS patients have premicroalbuminuria (Albumin/Creatinine Ratio (ACR) >7 mg/g) and is associated with metabolic syndromes (Patel et al., 2008; Caglar et al., 2011; Ziaee et al., 2012). In PCOS animal models, there is a high risk of age‐related chronic kidney injury (Patil et al., 2017). However, until now, there have been no extensive clinical or experimental studies looking into the association between female CKD patients and PCOS or vice versa. Furthermore, the underlying mechanisms are poorly understood.

In this study, we report that serum testosterone has a significantly positive correlation with the disease state of PCOS. Furthermore, serum testosterone expression is positively correlated with urinary excretions of κ‐light chains (KapU), λ‐light chains (LamU), α1‐microglobulin (α1‐MU), and β2‐microglobulin (β2‐MU), the markers of tubular proteinuria (Yu et al., 1983; Knudsen et al., 2000; Nasseh et al., 2016; Koratala et al., 2017; Ying et al., 2019). We also show that the follicular fluid (FF) from PCOS patients induces fibrotic lesions in renal tubular cells. Thus, we conclude that serum testosterone is a promising indicator of severe PCOS and highly associated with the incidence of kidney diseases in PCOS.

## Materials and Methods

### Patients

This study was approved by the Ethical Committee Faculty of Nanfang Hospital, Southern Medical University, and informed consents were obtained from all participants recruited into this study. The study was carried out in accordance with the World Medical Association Declaration of Helsinki. All methods were performed in accordance with the relevant guidelines and regulations. All participants were recruited from the center of reproduction medicine in Nanfang Hospital. The participants were excluded if they were diagnosed with kidney‐related diseases or other diseases that would affect renal function, such as acute kidney injury, nephrotic syndrome, diabetes, and rheumatological conditions. The recruited subjects included 55 infertile women with PCOS, and 69 healthy women who served as controls. Testosterone was tested by an enhanced chemiluminescence immunoassay using a Vitros ECi analyzer. Granulosa cells (GCs) were obtained from additional five PCOS patients with hyperandrogenism, five PCOS patients with normal expression of testosterone and five control participants. FF was collected from the PCOS patients with hyperandrogenism and controls.

PCOS was diagnosed according to the 2003 Rotterdam Criteria, with at least two of the following symptoms: (1) oligomenorrhea/amenorrhea; (2) polycystic ovaries (PCO) on ultrasonography; (3) clinical and/or biochemical signs of hyperandrogenism (hirsutism or acne) and exclusion of other etiologies (such as Cushing’s syndrome, congenital adrenal hyperplasia or testosterone secreting tumors). Clinical features including menstrual cycle, the number of antral follicles (AFC), body mass index (BMI), mean arterial pressure (MAP) as well as endocrine and biochemical parameters (on days 2−4 of the menstrual cycle) were recorded. The demographic and clinical data of the patients with PCOS are presented in Tables S1−S3.

### Urine collection and analysis

One hundred and twenty‐four urine samples from 55 PCOS patients and 69 control subjects were collected before participants were treated with assistant reproductive technology. The urine samples were centrifuged at 3000*g* for 10 min, and supernatants were obtained and stored at −80°C until further analysis. The urinary albumin to creatinine ratio (UACR), KapU, LamU, *α*1‐MU, *β*2‐MU, and urinary creatinine (UCr) were measured by automatic‐specific protein analyzers (AU480; Beckman Coulter, Pasadena, CA) in National Clinical Research Center of Kidney Disease in Nanfang Hospital, Southern Medical University. The kits were purchased from SIMENS9. The value of the UACR as well as KapU, LamU, α1‐MU, and β2‐MU were normalized to UCr.

### FF collection and primary cultured human ovary granulosa cells (GCs)

An antagonist protocol for controlled ovarian hyperstimulation (COH) was used in the cycle of *in vitro* fertilization and embryo transfer. In brief, ovarian hyperstimulation was achieved with the use of recombinant follicle‐stimulating hormone (FSH) from the third day of menstruation and adjusted as needed according to the ovarian response. Gonadotropin‐releasing hormone antagonist (GnRHant) was started in patients until they were triggered after the fifth day of COH. Transvaginal ultrasound‐guided oocyte retrieval was performed 35 h after the injection of human chorionic gonadotropin (hCG). The follicular fluid (FF) samples were obtained from the first‐punctured follicle (18−25 mm in diameter) during the oocyte aspiration. The samples were then centrifuged at 800*g* for 10 min, and the supernatants were stored at −80°C until further analysis. The testosterone expression in FF was analyzed. The GCs were isolated from FF obtained during oocyte retrieval for IVF from each subject. Briefly, FF was first centrifuged at 400*g* for 10 min. The pellet was resuspended with PBS, and then layered on 50% Percoll and centrifuged at 400*g* for 20 min. The volume ratio between PBS and 50% Percoll was 3:2. Three layers could then be distinguished, and the middle white layer was collected and washed with PBS at 600 g for 8 min at least twice. One milliliter of TRIzol reagent was added to the collected GCs (Life Technologies, Grand Island, NY) until RNA analysis.

### Human proximal tubular epithelial cell (HKC‐8) treatment

HKC‐8 cells, a human kidney proximal tubular cell line, were cultured in DMEM–Ham’s F12 medium containing 10% fetal bovine serum. HKC‐8 cells were treated with DMEM medium containing 10% FF for 24 h. The cells were then collected for various analyses.

### Quantitative reverse transcription‐polymerase chain reaction (qRT‐PCR)

The mRNA expression levels of caspase 3 and NLRP3 in GCs were analyzed by qRT‐PCR. Total RNA was extracted with TRIzol reagent (Life Technologies, Grand Island, NY) and cDNA was synthesized with the Reverse Transcription System kit (Promega, Madison, WI). qRT‐PCR was performed using SYBR Green PCR MasterMix (Applied Biosystems), according to the kit instructions by an ABI PRISM 7000 Sequence Detection System (Applied Biosystems, Foster City, CA). The PCR reaction system contained 12.5µL SYBR Green PCR MasterMix (Applied Biosystems), 5µL diluted RT product (1:10), and 0.5 mmol/L sense and antisense primer sets. The expression levels of various genes were determined by the comparative CT method (2^−ΔΔ^CT). Relative levels of mRNA were reported after normalization with β‐actin. The composition of the primers was as follows: caspase3: forward 5’‐GAAATTGTGGAATTGATGCGTGA‐3’, reverse 5’‐CTACAACGATCCCCTCTGAAAAA‐3’; NLRP3: forward 5’‐CGTGAGTCCCATTAAGATGGAGT‐3’, reverse 5’‐CGACCCTGTCCCTCAAATCC‐3’; actin: forward 5’‐CTCACCATGGATGATGATATCGC‐3’, 5’‐AGGAATCCTTCTGACCCATGC‐3’.

### Protein extracts and western blot

Total protein was extracted from HKC‐8 cells in lysis buffer containing 1% NP40, 0.1% SDS, 1 mg/mL PMSF, 1% protease inhibitor cocktail, and 1% phosphatase I and II inhibitor cocktail (Sigma). After separation on 10% SDS‐PAGE gels, the protein was transferred to membranes, which were later incubated with primary antibodies: anti‐fibronectin (F3648; Sigma‐Aldrich), anti‐α‐SMA (A2547; Sigma), and α‐tubulin (RM2007; Ray antibody Biotech, Beijing, China) overnight at 4°C. The signals were visualized using the enhanced chemiluminescence system (ECL, Amersham). Relative protein abundance was calculated after normalizing with α‐tubulin.

### Immunofluorescence staining

HKC‐8 cells were cultured on coverslips and fixed with 4% paraformaldehyde for 15 min at room temperature and immersed in 0.2% Triton X‐100 for 10 min. After blocking with 10% donkey serum for 30 min, the cells were immunostained with primary antibodies against fibronectin (F3648; Sigma‐Aldrich, St. Louis, MO) and a Cy3‐conjugated secondary antibody (Jackson ImmunoResearch Laboratories, West Grove, PA). Photos were taken with fluorescence microscopy (Leica DMi8; Leica Microsystems, Buffalo Grove, IL).

### Statistical analyses

Continuous variables are shown as the means ± SD, and categorical variables are expressed as *n* (%). Student’s t test and the Mann–Whitney U test were used to compare normally and non‐normally distributed variables, respectively. Correlations were conducted using Spearman’s correlation coefficient for non‐normally distributed data. Continuous variables among groups were compared using one‐way analysis of variance, followed by the LSD test. Statistical analyses were conducted with SPSS 22.0 software. A value of *P* < 0.05 was considered statistically significant.

## Results

### Subject characteristics

A total of 124 participants aged between 23 and 40 were enrolled. Among 55 patients with PCOS, hyperandrogenism (serum testosterone> 0.481 ng/mL is the diagnostic criteria in our hospital) was observed in 24 (43.64%) patients.

Two PCOS patients (3.64%) showed a slight increase in blood pressure (BP was 141/91 mmHg and 139/91 mmHg, respectively) and higher BMI index (BMI was 31 and 27, respectively). While the blood pressure in each of 69 controls was normal. One control subject showed slight increases in fasting glucose (GLU) (6.42 mmol/L) and BMI index 26). None was diagnosed hyperglycemia among 55 PCOS patients. Due to the small proportion of participants with the abnormal rate of BP and GLU, hierarchical analysis was not performed. The groups were matched in terms of age. Whereas, the BMI, length of the menstrual cycle, number of total antral follicles (No. of AFC‐Total), basal levels of luteinizing hormone (LH), estradiol (E_2_), and testosterone (T) in the PCOS group were significantly higher compared to controls. There were no significant differences in GLU, FSH, and prolactin (PRL) between two groups (Table 1).

**Table 1:** Clinical characteristics of participants.

Characteristic	Control (*N* = 69)	PCOS (*N* = 55)	*P‐*value
Age (y)	30.46 ± 4.31	29.09 ± 3.88	0.068
BMI (kg/m^2^)	22.03 ± 2.87	23.40 ± 3.80	0.029*
GLU (mmol/L)	5.07 ± 0.46	5.01 ± 0.47	0.497
Length of the menstrual cycle (d)	30.19 ± 2.56	67.91 ± 39.63	0.000**
No. of AFC‐Total	14.35 ± 4.26	27.47 ± 8.14	0.000**
Basal FSH (mIU/mL)	6.97 ± 1.40	6.44 ± 2.06	0.093
Basal LH (mIU/mL)	5.03 ± 1.75	10.22 ± 6.56	0.000**
Basal E_2_ (pg/mL)	34.10 ± 15.46	47.55 ± 40.13	0.022*
Basal T (ng/mL)	0.22 ± 0.08	0.45 ± 0.17	0.000*
Basal PRL (ng/mL)	18.58 ± 8.91	17.48 ± 6.50	0.442
MAP (mmHg)	85.70 ± 7.58	88.08 ± 8.24	0.096

Continuous variables are expressed as the mean ± SD. PCOS, polycystic ovary syndrome; BMI, body mass index; GLU, fasting glucose; FSH, follicle‐stimulating hormone; LH, luteinizing hormone; E2, estradiol; T, testosterone; PRL, prolactin; No. of AFC‐Total, the number of antral follicles with a diameter from 2 to 9 mm both in the left and right ovary; MAP, mean arterial pressure.

* *P* < 0.05.

** *P* < 0.01.

### Assessment of kidney function

**Figure 1:**
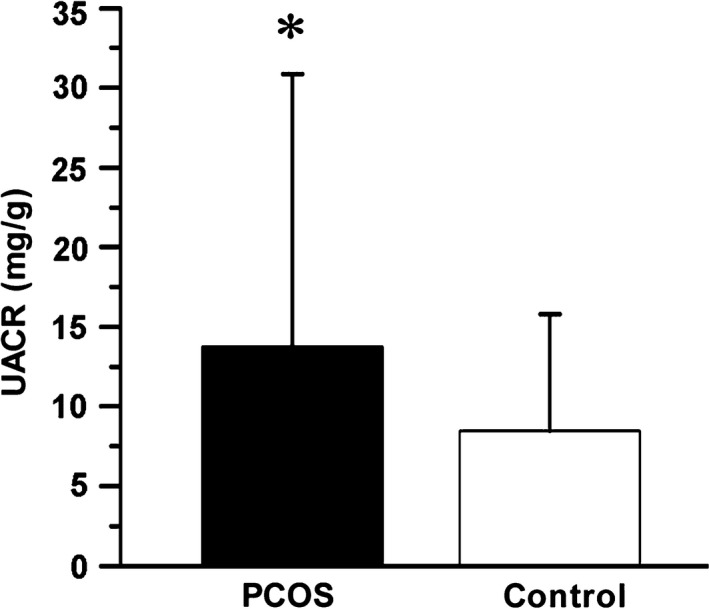
UACR is increased in PCOS group. PCOS, polycystic ovary syndrome; UACR, urinary albumin to creatinine ratio. *P < 0.05 versus controls (*n* = 55 in PCOS group, *n* = 69 in control group).

Previous studies have shown that PCOS links to abnormalities including obesity, DM, and cardiovascular disease. Notably, all these abnormalities are major risk factors for the progression of CKD (Castro and Coresh, 2009; Chadban et al., 2010; Teede et al., 2010; Cooney and Dokras, 2018). To analyze the correlation between PCOS and kidney injury, we examined kidney function through testing serum creatinine (SCr) and urea nitrogen (BUN), and proteinuria in all participants. As shown in Table 2 and Figure 1, the level of UACR was significantly higher (*P* < 0.05) in PCOS patients compared with controls. However, SCr and BUN showed no significant difference. Although no significant differences in the levels of KapU, LamU, α1‐MU, and β2‐MU, there was a rising trend for these tubular proteinuria markers in PCOS patients. Additionally, the proportion of patients with UACR higher than 7 mg/g was 45.45% in PCOS patients and 44.93% in controls.

**Table 2:** Assessment of kidney function in PCOS and control subjects.

Characteristic	Control (*N* = 69)	PCOS (*N* = 55)	*P‐*value
SCr (μmoI/L)	53.87 ± 7.87	54.69 ± 8.87	0.587
BUN (mmol/L)	4.26 ± 0.97	4.14 ± 1.10	0.523
UACR (mg/g)	8.47 ± 7.31	13.77 ± 17.10	0.043*
KapU (μg/mg)	9.38 ± 7.34	10.51 ± 11.22	0.499
LamU (μg/mg)	5.17 ± 4.06	5.48 ± 5.26	0.708
α1‐MU (μg/mg)	7.33 ± 5.39	7.80 ± 6.72	0.661
β2‐MU (μg/mg)	0.29 ± 0.23	0.30 ± 0.24	0.876
UACR> 7mg/g	44.93%	45.45%	0.953

SCr, Serum creatinine; BUN, blood urea nitrogen; UACR, urinary albumin to creatinine ratio; KapU, human immunoglobulin/light chain κ‐type; LamU, human immunoglobulin/light chain λ‐type; α1‐MU, α1‐Microglobulin; β2‐MU, β2‐Microglobulin.

* indicates *P* < 0.05

### Testosterone is correlated with the severity of PCOS

**Figure 2:**
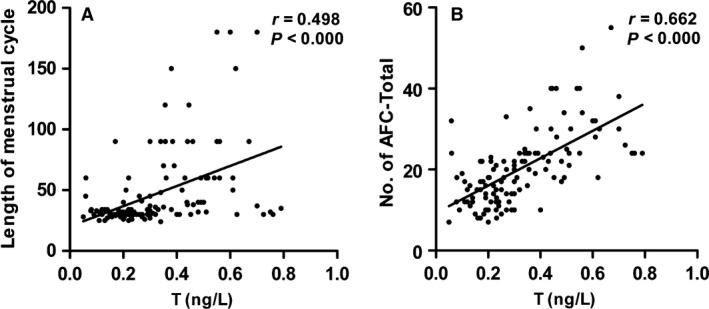
Serum testosterone (T) is correlated with the severity of PCOS. (A) Correlations between the level of T and length of the menstrual cycle (*n* = 124). (B) Correlations between the level of T and No. of AFC‐Total (*n* = 124). T, testosterone; No. of AFC‐Total, the number of antral follicles with a diameter from 2 to 9 mm both in the left and right ovary.

PCOS could be defined by multiple factors, including menstrual irregularity, polycystic ovary morphology (PCOM), and metabolic dysfunction. Notably, PCOS patients often complicate with hyperandrogenism. To further clarify the responsibility of testosterone for the disease severity of PCOS, we analyzed the correlation between serum testosterone and the length of the menstrual cycle or number of total antral follicles (No. of AFC‐Total). We also assessed the association of testosterone with BMI, MAP, and GLU, the three metabolic symptoms that commonly display in PCOS. As shown in Table 3 and Figure. 2A and B, serum testosterone positively correlated with the length of the menstrual cycle in 124 participants. Consistently, serum testosterone also had a significantly positive correlation with No. of AFC‐Total in both groups and showed an especially intimate correlation in PCOS patients (*P* < 0.01) (Table 3). However, as shown in Table 3, there was no correlation between serum testosterone and BMI, MAP, or GLU in two groups.

**Table 3:** Correlation analysis between serum testosterone (T) and the severity of PCOS in participants.

T				
Characteristic	Control (*N* = 69)		PCOS (*N* = 55)	
	*r*	*P*‐value	*r*	*P*‐value
BMI	−0.152	0.214	0.128	0.351
Length of the menstrual cycle	0.009	0.944	−0.074	0.59
No. of AFC‐Total	0.244	0.043*	0.387	0.003**
MAP	−0.078	0.526	0.249	0.067
GLU	0.131	0.284	−0.078	0.569

PCOS, polycystic ovary syndrome; T, testosterone; BMI, body mass index; No. of AFC‐Total, the number of antral follicles with the diameter from 2 to 9 mm both in the left and right ovary; GLU, fasting glucose; MAP: mean arterial pressure.

* *P* < 0.05.

** *P* < 0.01.

### Testosterone is correlated with tubular injury in PCOS with hyperandrogenism

**Figure 3:**
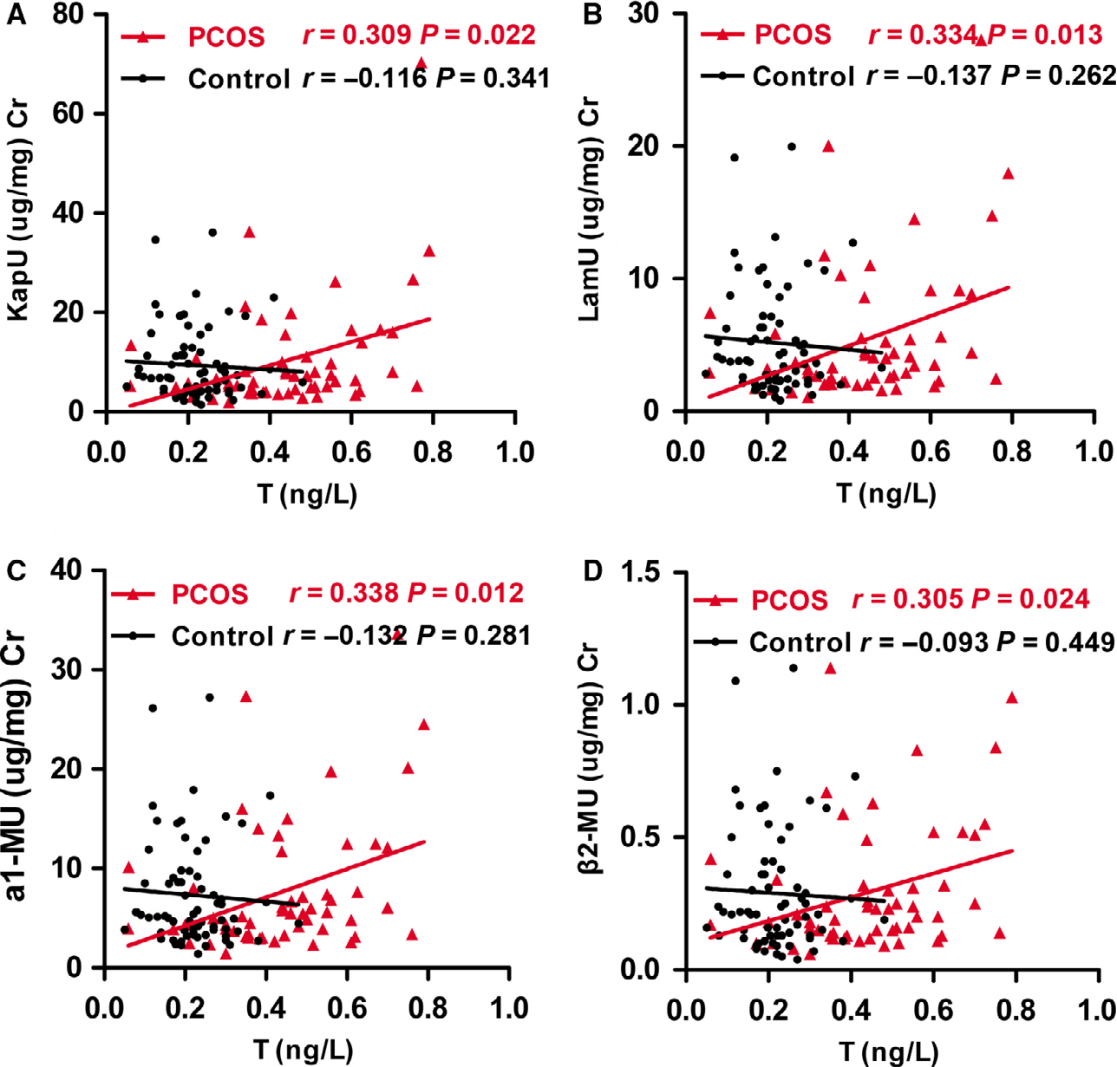
Correlation analysis between serum testosterone and tubular proteinuria in PCOS patients and controls, respectively. (A) Correlation between the level of T and KapU in PCOS patients and controls, respectively. (B) Correlation between the level of T and LamU in PCOS patients and controls, respectively. (C) Correlation between the level of T and a1‐MU in PCOS patients and controls, respectively. (D) Correlation between the level of T and β2‐MU in PCOS patients and controls, respectively. PCOS, polycystic ovary syndrome; KapU, human immunoglobulin/light chain κ‐type; LamU, human immunoglobulin/light chain λ‐type; α1‐MU, α1‐microglobulin; β2‐MU, β2‐microglobulin.

To identify the role of serum testosterone in PCOS‐associated kidney injury, we performed Spearman’s correlation analysis in two groups. First, the correlation between serum testosterone and UACR in two groups was analyzed. There was no correlation between them in two groups (data not shown). We next assessed the urinary levels of KapU, LamU, α1‐MU, and β2‐MU, which represent the proteinuria originated from renal tubular epithelia cell injury. All of them were performed correlation test with serum testosterone. As shown in Table 4 and Figure 3A–D, in PCOS patients, serum testosterone had a significantly positive correlation with all of these tubular proteinuria markers. These suggest testosterone plays a critical role in PCOS‐associated kidney injury, especially in tubular epithelial cell injury.

**Table 4:** Correlation analysis between serum testosterone (T) and tubular proteinuria markers in participants.

T				
Characteristic	Control (*N* = 69)		PCOS (*N* = 55)	
	*r*	*P*‐value	*r*	*P*‐value
KapU (μg/mg)	−0.116	0.341	0.309	0.022*
LamU (μg/mg)	−0.137	0.262	0.334	0.013*
a1‐MU (μg/mg)	−0.132	0.281	0.338	0.012*
β2‐MU (μg/mg)	−0.093	0.449	0.305	0.024*

PCOS, polycystic ovary syndrome; T, testosterone; KapU, human immunoglobulin/light chain κ‐type; LamU, human immunoglobulin/light chain λ‐type; a1‐MU, a1‐microglobulin; β2‐MU, β2‐microglobulin.

* *P* < 0.05

### Testosterone induces apoptosis and inflammation in primary cultured GCs

**Figure 4:**
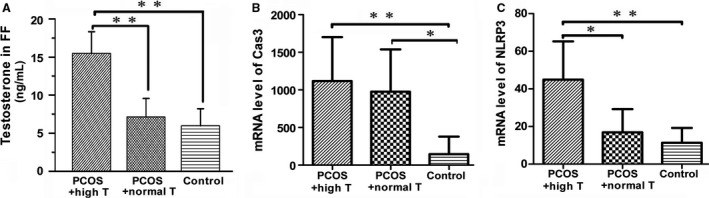
Testosterone and mRNA expression levels of caspase‐3 and NLRP3 in isolated human GCs. (A) The testosterone (T) level (ng/ml) in FF from different groups as indicated. (B) The mRNA expression of caspase‐3 (Cas3) in GCs from PCOS patients with high and normal testosterone, and control subjects. (C)The mRNA expression of NLRP3 in GCs in different groups. *P < 0.05, **P < 0.01; T, testosterone; PCOS, polycystic ovary syndrome; PCOS + high T, PCOS with hyperandrogenism; PCOS + normal T, PCOS patients with non‐hyperandrogenism.

To further confirm the effects of testosterone on PCOS injury, we cultured primary GCs. The cells were collected from the follicular fluid (FF) samples of five PCOS patients with hyperandrogenism (with high testosterone), five PCOS patients with non‐hyperandrogenism (with normal testosterone) and five healthy controls. First, the levels of testosterone in FF were analyzed (Fig. 4A). We next examined the mRNA levels of the apoptosis‐related factor caspase‐3 and inflammation‐related factor NLRP3 by qRT‐PCR. As shown in Figure 4B, compared with control group, the mRNA level of caspase‐3 in PCOS groups significantly increased. The similar results were observed when NLRP3 was assessed (Fig. 4C). It is notable that the expression of caspase 3 and NLRP3 in GCs from PCOS patients with high testosterone showed more statistical difference (*P* < 0.01), suggesting the important role of testosterone in GCs injury.

### FF from PCOS patients with hyperandrogenism induces fibrotic injury in cultured HKC‐8 cells

**Figure 5:**
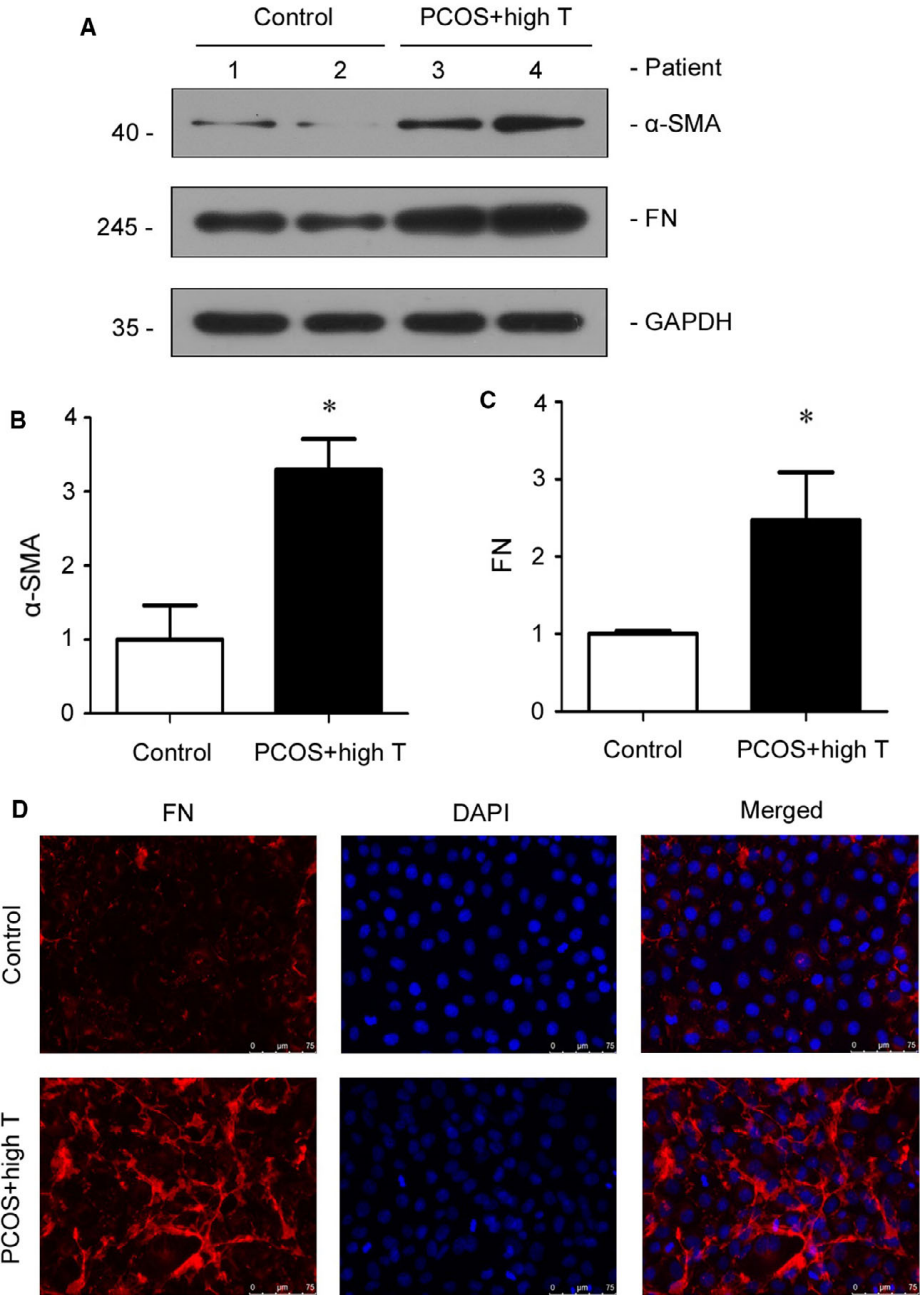
FF from PCOS patients with hyperandrogenism induces fibrotic injury in cultured HKC‐8 cells. (A) Representative western blots show the expression of α‐SMA and fibronectin (FN). HKC‐8 cells were stimulated with DMEM medium containing 10% of FF for 24 h. Whole‐cell lysates were analyzed by western blot. Quantitative data of α‐SMA (B) and FN (C) are shown. *P < 0.05 versus control group (*n* = 3). (D) Representative immunofluorescence staining show FN expression in HKC‐8 cells. HKC‐8 cells were cultured on coverslips and then stimulated with FF from PCOS patients with hyperandrogenism or controls for 24 h. The immunofluorescence of FN was detected by an antibody against FN (red) and counterstained with DAPI (blue). α‐SMA, α‐smooth muscle actin; FN, fibronectin; FF, follicular fluid; T, testosterone; PCOS, polycystic ovary syndrome; PCOS + high T, PCOS with hyperandrogenism.

To further identity the correlation between PCOS and kidney injury, we cultured HKC‐8 cells and treated cells with FF from PCOS patients with hyperandrogenism and healthy controls. The fibrotic injury markers of fibronectin and α‐SMA were assessed by western blot analyses. As shown in Figure 5A–C, the FF from PCOS patients with hyperandrogenism could significantly induce the upregulation of fibronectin and α‐SMA in HKC‐8 cells. The similar results were observed when fibronectin was detected by immunofluorescence staining (Fig. 5D). Taken together, these data further implicate that PCOS is highly associated with kidney tubular cell injury. Serum testosterone plays a critical role in mediating PCOS‐induced kidney fibrosis.

## Discussion

PCOS has a close association with the progression of metabolic abnormalities such as obesity, diabetes and hypertension, which are the major causes of kidney disease (Castro and Coresh, 2009; Chadban et al., 2010; Cooney and Dokras, 2018; Teede et al., 2018). These suggest PCOS may have an intimate association with kidney injury. Few studies have investigated the underlying mechanism of PCOS‐associated kidney injury. In this study, we have established a link of PCOS with kidney injury through clinical and experimental studies.

To test the hypothesis that PCOS is associated with kidney injury, we evaluated kidney function in patients with PCOS and controls. We first assessed UACR, which refers to the ratio of microalbuminuria to creatinine in urine. An increase in UACR often indicates glomerular injury (Zylka et al., 2018). In PCOS patients, the level of UACR was significantly higher (Table 2, Fig. 1), suggesting a close association between PCOS and kidney injury. Consistent with our findings, previous reports indicate there is an association between PCOS and albuminuria (Patel et al., 2008; Ziaee et al., 2012). We also found that not all PCOS patients exhibited an increased UACR, and the proportion of patients with the level of UACR> 7 mg/g was 45.45% in PCOS group and 44.93% in control group, which is consistent with the other reports (Patel et al., 2008; Ziaee et al., 2012).

We next explored the underlying mechanism. To address the pathogenesis, we first analyzed serum testosterone, the most critical marker in PCOS patients. Similar to previous reports, PCOS patients had a high incidence of hyperandrogenism (43.64%) in our study. We then explored the association between serum testosterone and the disease severity of PCOS. Abnormality of the menstrual cycle is one of the critical characteristics in PCOS patients. The prolongation of the menstrual cycle could result in the high frequency of anovulation, which would lead to amenorrhea, infertility, endometrial hyperproliferation and even carcinogenesis (Barry et al., 2014). These can eventually cause the more serious conditions of PCOS. Polycystic ovary (PCO) is another critical feature in PCOS patients, which defined by the presentations of at least 12 antral follicles (AFC) with a diameter from 2 to 9 mm in the whole ovary and/or an ovarian volume over 10 mL (Balen et al., 2003). The disease state of PCOS is highly associated with the increase in the numbers of AFC. Excessive AFC could secret estrogen, which inhibits the secretion of FSH by the negative feedback of the gonadal axis. These manifestations cooperatively lead to anovulation. Therefore, the increase in AFC could indirectly reflect the severity of PCOS disease. According to the theory mentioned above, we adopted the menstrual cycle and AFC to assess the severity of PCOS disease state. Not surprisingly, we found that the level of serum testosterone was positively correlated with the length of the menstrual cycle and the total numbers of AFC in both the right and left ovaries, suggesting that testosterone can promote the development and progression of PCOS (Table 3, Fig. 2). This is consistent with other studies (Jonard and Dewailly, 2004). We also analyzed the correlation between testosterone and BMI, MAP, and GLU, and found no correlation (Table 3). The reason may be related to the ethnic variations. For example, in China, there are more PCOS patients with menstrual disorders and PCO, but few of them complicate with obesity.

Other studies have revealed testosterone in the FF in PCOS patients is higher than that in controls (Qu et al., 2010). To further claim the role of testosterone in the pathogenesis of PCOS, we collected the FF from PCOS patients with serum testosterone at different levels. It is well‐known that FF is a growing microenvironment for GCs and oocytes. Hence, we isolated GCs from it to check the cellular injury. We first assessed the expression of caspase‐3, an important enzyme‐initiating cell apoptosis. We found that caspase‐3 expression was significantly higher in PCOS participants (Fig. 4). Similar to our results, previous studies have shown that testosterone can induce apoptosis and the upregulation of caspase‐3 in mouse GCs (Zhao et al., 2013). We next analyzed NLRP3, an inflammasome which plays an important role in the pathogenesis of diabetes, cardiovascular, and kidney inflammatory injury through reactivating multiple inflammatory responses including apoptosis (Takahashi, 2014; Volpe et al., 2016; Romero et al., 2017). Our results showed that the mRNA expression level of NLRP3 in GCs was significantly higher in PCOS with hyperandrogenism group. Notably, there was also a significant difference between the hyperandrogenism and normal testosterone groups in PCOS patients (Fig. 4). These results further confirm the pivotal role of testosterone in loss of ovarian function.

We further investigated the role of testosterone in PCOS‐associated kidney injury. We performed correlation analyses of serum testosterone with the urinary excretion levels of KapU, LamU, α1‐MU, and β2‐MU, the markers of kidney tubular injury (Yu et al., 1983; Knudsen et al., 2000; Nasseh et al., 2016; Koratala et al., 2017; Ying et al., 2019). Our results showed that there was a significantly positive correlation between serum testosterone and these tubular proteinuria markers in PCOS patients, but there was no correlation in control subjects (Table 4, Fig. 3). This further demonstrates that testosterone plays an important role in the pathogenesis of PCOS‐associated kidney injury, especially tubular cell injury. To further test the detrimental role of testosterone in PCOS‐related renal tubular cell injury, we collected the FF from PCOS patients with hyperandrogenism and then treated HKC‐8 cells. Administration of FF from PCOS patients with hyperandrogenism significantly stimulated the fibrotic lesions, the important indicators of kidney tubular injury (Gewin, 2018), in HKC‐8 cells (Fig. 5). These results provide further evidence that testosterone may be one of the main mechanisms in mediating PCOS‐associated kidney injury. Consistently, previous studies have reported that testosterone could cause apoptosis in kidney tubulogenic cells (Verzola et al., 2004). Moreover, a meta‐analysis has shown that men have a greater incidence of kidney disease and higher risk to progress into end‐stage kidney failure than women (Neugarten et al., 2000). Therefore, we propose that testosterone can serve as an indicator reflecting PCOS and PCOS‐associated kidney injury. Although UACR was higher in PCOS patients than that in controls, we could not find a correlation between serum testosterone and UACR. The reasons maybe lie in the sample size of PCOS patients in our study is small, and more androgen receptors are localized in renal tubular epithelial cells than that in glomerular cells. More studies should be performed to testify the hypothesis in the future.

To the best of our knowledge, this is the first study that demonstrates a definitive relationship between PCOS and kidney injury. First, our clinical analysis showed that UACR is higher in PCOS patients than controls, suggesting that PCOS is closely associated with kidney injury. Second, we found that serum testosterone in PCOS patients are positively correlated with the urinary protein excretion levels of which reflect the injury in kidney tubules. These results demonstrate that testosterone plays an important role in PCOS‐associated kidney injury. Third, we analyzed the effects of FF from PCOS patients with hyperandrogenism on HKC‐8 cells. We found the upregulated fibrotic lesions in PCOS FF‐treated HKC‐8 cells, suggesting that testosterone mediates PCOS‐related kidney injury through promoting the fibrosis in tubular epithelial cells.

In this study, because mass spectrometry has not yet been established in our clinics, we tested the total testosterone in serum by the chemiluminescence immunoassay but not the “gold standard” testing method of testosterone status such as liquid chromatography‐tandem mass spectrometry. Future studies should be designed to analyze testosterone by multiple methods. Furthermore, a larger sample size including various ethnic backgrounds from different regions should be recruited to verify the conclusion and deeply explore the underlying mechanisms. Specially, studying a prospective cohort of women with PCOS and BMI more than 30 would be more meaningful to assess the association between PCOS and renal injury.

Although more studies are needed, these results provide proof of principle that serum testosterone plays a pivotal role in the onset and development of PCOS and PCOS‐associated kidney disease. Continued monitoring and early intervention of serum testosterone would be of great value to prevent the high risk of kidney injury for PCOS patients. Overall, serum testosterone may be regarded as a novel indicative marker for clinical prevention and treatment of PCOS and the related kidney diseases.

## Conflict of interest

The authors declare that there are no competing interests associated with the manuscript.

## Ethics approval

This study was approved by the Ethical Committee Faculty of Nanfang Hospital, Southern Medical University, and informed consent was obtained from all of the participants recruited into this study and was carried out in accordance with the World Medical Association Declaration of Helsinki. All methods were performed in accordance with the relevant guidelines and regulations. The number of ethical approvals is NFEC‐2018‐154.

## Supporting information




**Table S1.** Demographic characteristics of the participants in the study.
**Table S2.** Parameter values of control subjects and PCOS patients.
**Table S3.** Parameter values in isolated human granulosa cells.

## References

[phy214219-bib-0001] Azziz, R. , E. Carmina , Z. Chen , A. Dunaif , J. S. Laven , R. S. Legro , et al. 2016 Polycystic ovary syndrome. Nat. Rev. Dis. Primers 2:16057 10.1038/nrdp.2016.57 27510637

[phy214219-bib-0002] Balen, A. H. , J. S. Laven , S. L. Tan , and D. Dewailly . 2003 Ultrasound assessment of the polycystic ovary: international consensus definitions. Hum. Reprod. Update. 9:505–514. 10.1093/humupd/dmg044 14714587

[phy214219-bib-0003] Barry, J. A. , M. M. Azizia , and P. J. Hardiman . 2014 Risk of endometrial, ovarian and breast cancer in women with polycystic ovary syndrome: a systematic review and meta‐analysis. Hum. Reprod. Update 20:748–758. 10.1093/humupd/dmu012 24688118 PMC4326303

[phy214219-bib-0004] Behboudi‐Gandevani, S. , M. Amiri , Y. R. Bidhendi , M. Noroozzadeh , M. Farahmand , M. Rostami Dovom , et al. 2018 The risk of metabolic syndrome in polycystic ovary syndrome: A systematic review and meta‐analysis. Clin. Endocrinol. 88:169–184. 10.1111/cen.13477 28930378

[phy214219-bib-0005] Caglar, G. S. , E. Oztas , D. Karadag , R. Pabuccu , and A. A. Eren . 2011 The association of urinary albumin excretion and metabolic complications in polycystic ovary syndrome. Eur. J. Obstet. Gynecol. Reprod. Biol. 154:57–61. 10.1016/j.ejogrb.2010.08.024 20888116

[phy214219-bib-0006] Castro, A. F. , and J. Coresh . 2009 CKD surveillance using laboratory data from the population‐based National Health and Nutrition Examination Survey (NHANES). Am. J. Kidney Dis. 53:S46–S55. 10.1053/j.ajkd.2008.07.054 19231761 PMC2677815

[phy214219-bib-0007] Chadban, S. , M. Howell , S. Twigg , M. Thomas , G. Jerums , A. Cass , et al. 2010 The CARI guidelines. Assessment of kidney function in type 2 diabetes. Nephrology 15(Suppl 1):S146–S161. 10.1111/j.1440-1797.2010.01239.x 20591027

[phy214219-bib-0008] Cooney, L. G. , and A. Dokras . 2018 Beyond fertility: polycystic ovary syndrome and long‐term health. Fertil. Steril. 110:794–809. 10.1016/j.fertnstert.2018.08.021 30316414

[phy214219-bib-0009] Cooney, L. G. , I. Lee , M. D. Sammel , and A. Dokras . 2017 High prevalence of moderate and severe depressive and anxiety symptoms in polycystic ovary syndrome: a systematic review and meta‐analysis. Hum. Reprod. 32:1075–1091. 10.1093/humrep/dex044 28333286

[phy214219-bib-0010] Gewin, L. S. 2018 Kidney fibrosis: primacy of the proximal tubule. Matrix. Biol. 68–69:248–262. 10.1016/j.matbio.2018.02.006 PMC6015527 29425694

[phy214219-bib-0011] Jakubowicz, D. , M. Barnea , J. Wainstein , and O. Froy . 2013 Effects of caloric intake timing on insulin resistance and hyperandrogenism in lean women with polycystic ovary syndrome. Clin. Sci. 125:423–432. 10.1042/CS20130071 23688334

[phy214219-bib-0012] Jonard, S. , and D. Dewailly . 2004 The follicular excess in polycystic ovaries, due to intra‐ovarian hyperandrogenism, may be the main culprit for the follicular arrest. Hum. Reprod. Update. 10:107–117. 10.1093/humupd/dmh010 15073141

[phy214219-bib-0013] Kahal, H. , I. Kyrou , A. A. Tahrani , and H. S. Randeva . 2017 Obstructive sleep apnoea and polycystic ovary syndrome: a comprehensive review of clinical interactions and underlying pathophysiology. Clin. Endocrinol. 87:313–319. 10.1111/cen.13392 28640938

[phy214219-bib-0014] Knudsen, L. M. , M. Hjorth , and E. Hippe . 2000 Renal failure in multiple myeloma: reversibility and impact on the prognosis. Nordic Myeloma Study Group. Eur. J. Haematol. 65:175–181. 11007053 10.1034/j.1600-0609.2000.90221.x

[phy214219-bib-0015] Koratala, A. , A. A. Ejaz , W. M. Hiser , and W. L. Clapp .2017 Trifecta of light chain cast nephropathy, monoclonal plasma cell infiltrates, and light chain proximal tubulopathy. Kidney Int. 92:1559 10.1016/j.kint.2017.08.010 29153147

[phy214219-bib-0016] Li, R. , Q. Zhang , D. Yang , S. Li , S. Lu , X. Wu , et al. 2013 Prevalence of polycystic ovary syndrome in women in China: a large community‐based study. Hum Reprod 28:2562–2569. 10.1093/humrep/det262 23814096

[phy214219-bib-0017] Lizneva, D. , L. Suturina , W. Walker , S. Brakta , L. Gavrilova‐Jordan , and R. Azziz . 2016 Criteria, prevalence, and phenotypes of polycystic ovary syndrome. Fertil. Steril. 106:6–15. 10.1016/j.fertnstert.2016.05.003 27233760

[phy214219-bib-0018] Nasseh, H. , S. Abdi , A. Roshani , and E. Kazemnezhad . 2016 Urinary Beta‐2Microglobulin: An Indicator of Kidney Tubular Damage after Extracorporeal Shock Wave Lithotripsy. Urol. J. 13:2911–2915. 27928813

[phy214219-bib-0019] Neugarten, J. , A. Acharya , and S. R. Silbiger . 2000 Effect of gender on the progression of nondiabetic kidney disease: a meta‐analysis. J. Am. Soc. Nephrol. 11:319–329. 10665939 10.1681/ASN.V112319

[phy214219-bib-0020] Nigam, S. K. , and K. T. Bush . 2019 Uraemic syndrome of chronic kidney disease: altered remote sensing and signalling. Nat. Rev. Nephrol. 15:301–316. 30728454 10.1038/s41581-019-0111-1 PMC6619437

[phy214219-bib-0021] Patel, A. A. , Z. T. Bloomgarden , and W. Futterweit . 2008 Premicroalbuminuria in women with polycystic ovary syndrome: a metabolic risk marker. Endocr. Pract. 14:193–200. 10.4158/EP.14.2.193 18308657

[phy214219-bib-0022] Patil, C. N. , L. C. Racusen , and J. F. Reckelhoff . 2017 Consequences of advanced aging on kidney function in chronic hyperandrogenemic female rat model: implications for aging women with polycystic ovary syndrome. Physiol Rep 5: pii: e13461. 29051304 10.14814/phy2.13461 PMC5661229

[phy214219-bib-0023] Peterson, B. D. , C. S. Sejbaek , M. Pirritano , and L. Schmidt . 2014 Are severe depressive symptoms associated with infertility‐related distress in individuals and their partners? Hum. Reprod. 29:76–82. 10.1093/humrep/det412 24256990

[phy214219-bib-0024] Piccoli, G. B. , M. Alrukhaimi , Z. H. Liu , E. Zakharova , and A. Levin , World Kidney Day Steering Committee . 2018 Women and kidney disease: reflections on World Kidney Day. 2018. Kidney Health and Women's Health: a case for optimizing outcomes for present and future generations. Nephrol Dial Transpl 33:189–193. 10.1093/ndt/gfx358 29401358

[phy214219-bib-0025] Qu, F. , F. F. Wang , X. E. Lu , M. Y. Dong , J. Z. Sheng , P. P. Lv , et al. 2010 Altered aquaporin expression in women with polycystic ovary syndrome: hyperandrogenism in follicular fluid inhibits aquaporin‐9 in granulosa cells through the phosphatidylinositol 3‐kinase pathway. Hum. Reprod. 25:1441–1450. 10.1093/humrep/deq078 20378617

[phy214219-bib-0026] Reis, A. M. , and K. Honorato‐Sampaio . 2018 C‐type natriuretic peptide: a link between hyperandrogenism and anovulation in a mouse model of polycystic ovary syndrome. Clin. Sci. 132:905–908. 10.1042/CS20171491 29739821

[phy214219-bib-0027] Romero, C. A. , A. Remor , A. Latini , A. L. De Paul , A. I. Torres , and J. H. Mukdsi . 2017 Uric acid activates NRLP3 inflammasome in an in‐vivo model of epithelial to mesenchymal transition in the kidney. J. Mol. Histol. 48:209–218. 10.1007/s10735-017-9720-9 28374152

[phy214219-bib-0028] Santoro, N. , E. Eisenberg , J. C. Trussell , L. B. Craig , C. Gracia , H. Huang , et al. 2016 Fertility‐related quality of life from two RCT cohorts with infertility: unexplained infertility and polycystic ovary syndrome. Hum Reprod 31:2268–2279. 10.1093/humrep/dew175 27402910 PMC5027926

[phy214219-bib-0029] Showell, M. G. , R. Mackenzie‐Proctor , V. Jordan , R. Hodgson , and C. Farquhar . 2018 Inositol for subfertile women with polycystic ovary syndrome. Cochrane Database Syst. Rev. 12: CD012378: 10.1002/14651858.CD012378.pub2 PMC6516980 30570133

[phy214219-bib-0030] Takahashi, M. 2014 NLRP3 inflammasome as a novel player in myocardial infarction. Int. Heart J. 55:101–105. 10.1536/ihj.13-388 24632952

[phy214219-bib-0031] Teede, H. , A. Deeks , and L. Moran . 2010 Polycystic ovary syndrome: a complex condition with psychological, reproductive and metabolic manifestations that impacts on health across the lifespan. BMC Med 8:4 10.1186/1741-7015-8-41. 20591140 PMC2909929

[phy214219-bib-0032] Teede, H. J. , M. L. Misso , M. F. Costello , A. Dokras , J. Laven , L. Moran , et al. 2018 Recommendations from the international evidence‐based guideline for the assessment and management of polycystic ovary syndrome. Hum. Reprod. 33:1602–1618. 10.1093/humrep/dey256 30052961 PMC6112576

[phy214219-bib-0033] Verzola, D. , M. T. Gandolfo , F. Salvatore , B. Villaggio , F. Gianiorio , P. Traverso , et al. 2004 Testosterone promotes apoptotic damage in human kidney tubular cells. Kidney Int. 65:1252–1261. 10.1111/j.1523-1755.2004.00497.x 15086464

[phy214219-bib-0034] Volpe, C. M. , P. M. Anjos , and J. A. Nogueira‐Machado . 2016 Inflammasome as a new therapeutic target for diabetic complications. Recent Pat. Endocr. Metab. Immune Drug Discov. 10:56–62. 26899852 10.2174/1872214810666160219163314

[phy214219-bib-0035] Wang, X. , H. Wang , W. Liu , Z. Zhang , Y. Zhang , W. Zhang , et al. 2018 High level of C‐type natriuretic peptide induced by hyperandrogen‐mediated anovulation in polycystic ovary syndrome mice. Clin. Sci. 132:759–776. 10.1042/CS20171394 29535265

[phy214219-bib-0036] Yang, R. , S. Yang , R. Li , P. Liu , J. Qiao , and Y. Zhang . 2016 Effects of hyperandrogenism on metabolic abnormalities in patients with polycystic ovary syndrome: a meta‐analysis. Reprod. Biol. Endocrin. 14:67 10.1186/s12958-016-0203-8 PMC5069996 27756332

[phy214219-bib-0037] Ying, W. Z. , X. Li , S. Rangarajan , W. Feng , L. M. Curtis , and P. W. Sanders . 2019 Immunoglobulin light chains generate proinflammatory and profibrotic kidney injury. J. Clin. Invest. 129:2792–2806. 10.1172/JCI125517 31205024 PMC6597222

[phy214219-bib-0038] Yu, H. , Y. Yanagisawa , M. A. Forbes , and E. Kazemnezhad . 1983 Alpha‐1‐microglobulin: an indicator protein for kidney tubular function. J. Clin. Pathol. 36:253–259. 6186698 10.1136/jcp.36.3.253 PMC498194

[phy214219-bib-0039] Yu, H. F. , H. S. Chen , D. P. Rao , and J. Gong . 2016 Association between polycystic ovary syndrome and the risk of pregnancy complications: a PRISMA‐compliant systematic review and meta‐analysis. Medicine (Baltimore) 95:e4863 10.1097/MD.0000000000004863 28002314 PMC5181798

[phy214219-bib-0040] Zhao, K. K. , Y. G. Cui , Y. Q. Jiang , J. Wang , M. Li , Y. Zhang , et al. 2013 Effect of HSP10 on apoptosis induced by testosterone in cultured mouse ovarian granulosa cells. Eur. J. Obstet. Gynecol. Reprod. Biol. 171:301–306. 10.1016/j.ejogrb.2013.09.026 24161766

[phy214219-bib-0041] Ziaee, A. , S. Oveisi , A. Ghorbani , S. Hashemipour , and M. Mirenayat . 2012 Association between metabolic syndrome and premicroalbuminuria among Iranian women with Polycystic Ovary Syndrome: a case control study. Glob. J. Health Sci. 5:187–192. 10.5539/gjhs.v5n1p187 23283052 PMC4776989

[phy214219-bib-0042] Zylka, A. , P. Dumnicka , B. Kusnierz‐Cabala , A. Gala‐Błądzińska , P. Ceranowicz , J. Kucharz , et al. 2018 Markers of glomerular and tubular damage in the early stage of kidney disease in type 2 diabetic patients. Mediat. Inflamm. 2018:7659243 10.1155/2018/7659243 PMC6109534 30158836

