# Supplementary material for: Serum testosterone acts as a prognostic indicator in polycystic ovary syndrome‐associated kidney injury
Source: Physiol Rep. 2019 Aug 25;7(16):e14219. doi: 10.14814/phy2.14219 (PMC6709419; doi:10.14814/phy2.14219)
Supplement: Supplementary file 1 — Table S1. Demographic characteristics of the participants in the study. Table S2. Parameter values of control subjects and PCOS patients. Table S3. Parameter values in isolated human granulosa cells. [file PHY2-7-e14219-s001.docx]

**Supplementary Table 1. Demographic characteristics of the participants in the study**

| **Characteristic** | **Cohort** | | |
| --- | --- | --- | --- |
|  | **Controls (N=69)** | | **PCOS patients (N=55)** |
| **Age at entry--years** |  | |  |
| Mean±SD | 30±4 | | 29±4 |
| Range | 23-40 | | 23-40 |
| **Length of menstrual cycle-- days**** |  | |  |
| Mean±SD | 30±3 | | 68±40 |
| Range | 24-34 | | 30-180 |
| **No. Of AFC-Total** ** |  |  |  |
| Mean±SD | 14±4 | | 27±8 |
| Range | 7-23 | | 16-55 |
| **Basal serum testosterone-- ng/mL** |  | |  |
| Mean±SD | 0.22±0.08 | | 0.45±0.17 |
| Range | 0.05-0.48 | | 0.059-0.79 |
| **PCOS Diagnosis -- No. (%)†** |  | |  |
| oligomenorrhea / amenorrhoea | 0(0) | | 48(55) |
| polycystic ovaries (PCO) | 0(0) | | 54(55) |
| hyperandrogenism | 0(0) | | 24(55) |

*There were significant difference in length of menstrual cycle and No. Of AFC-Total between PCOS patients and control groups. The urinary UACR, KapU, LamU, α1-MU and β2-MU were measured from all participants.

**Supplementary Table 2. Parameter values of control subjects and PCOS patients**

| **all participants(N=124)** | | | | | | | |  |  |
| --- | --- | --- | --- | --- | --- | --- | --- | --- | --- |
| **No.** | **group** | **Age** | **UACR (mg/g)** | **KapU**  **(μg/mg)** | **LamU**  **(μg/mg)** | **α1-MU**  **(μg/mg)** | **β2-MU**  **(μg/mg)** | **Bp**  **(mmHg)** | **GLU**  **(mmol/L)** |
| 1 | PCOS | 28 | 70.77 | 36.25 | 20.03 | 27.35 | 1.14 | 112/78 | 4.80 |
| 2 | PCOS | 33 | 4.44 | 19.92 | 11.01 | 15.02 | .63 | 100/60 | 5.36 |
| 3 | PCOS | 26 | 4.33 | 7.24 | 4.00 | 5.46 | .23 | 129/79 | 4.63 |
| 4 | PCOS | 33 | 27.41 | 5.75 | 3.18 | 4.34 | .18 | 100/64 | 5.81 |
| 5 | PCOS | 40 | 4.19 | 4.14 | 2.29 | 3.12 | .13 | 133/78 | 4.69 |
| 6 | PCOS | 25 | 15.52 | 5.22 | 2.88 | 3.94 | .16 | 126/80 | 5.36 |
| 7 | PCOS | 25 | 5.94 | 3.54 | 1.96 | 2.67 | .11 | 108/66 | 5.74 |
| 8 | PCOS | 28 | 6.77 | 10.62 | 5.87 | 8.02 | .34 | 124/76 | 4.83 |
| 9 | PCOS | 30 | 4.74 | 3.72 | 2.06 | 3.06 | .12 | 120/80 | 6.02 |
| 10 | PCOS | 26 | 3.00 | 5.13 | 2.73 | 5.97 | .16 | 111/70 | 4.26 |
| 11 | PCOS | 33 | 17.59 | 1.93 | 1.07 | 1.46 | .06 | 116/78 | 4.85 |
| 12 | PCOS | 36 | 2.83 | 5.75 | 3.18 | 4.34 | .18 | 106/61 | 4.99 |
| 13 | PCOS | 26 | 8.39 | 2.81 | 1.56 | 4.20 | .09 | 100/60 | 5.35 |
| 14 | PCOS | 25 | 5.64 | 2.59 | 1.43 | 2.37 | .08 | 120/75 | 5.10 |
| 15 | PCOS | 26 | 4.83 | 3.01 | 1.67 | 2.48 | .10 | 112/78 | 4.50 |
| 16 | PCOS | 28 | 7.63 | 10.01 | 5.53 | 13.33 | .32 | 127/80 | 4.62 |
| 17 | PCOS | 34 | 28.71 | 4.95 | 1.73 | 3.88 | .10 | 125/82 | 4.22 |
| 18 | PCOS | 29 | 5.16 | 5.25 | 2.47 | 3.37 | .14 | 116/78 | 4.90 |
| 19 | PCOS | 25 | 17.97 | 26.72 | 14.77 | 20.16 | .84 | 112/78 | 4.60 |
| 20 | PCOS | 27 | 10.44 | 32.47 | 17.95 | 24.50 | 1.03 | 112/78 | 4.20 |
| 21 | PCOS | 30 | 3.46 | 5.27 | 2.68 | 3.71 | .15 | 113/78 | 5.09 |
| 22 | PCOS | 30 | 10.46 | 15.55 | 8.59 | 11.73 | .49 | 109/58 | 4.57 |
| 23 | PCOS | 25 | 3.32 | 21.25 | 11.75 | 16.03 | .67 | 113/65 | 4.51 |
| 24 | PCOS | 25 | 30.90 | 13.44 | 7.43 | 10.14 | .42 | 110/62 | 5.20 |
| 25 | PCOS | 29 | 5.74 | 8.00 | 4.42 | 6.03 | .25 | 115/75 | 4.60 |
| 26 | PCOS | 26 | - | 4.06 | 2.24 | 3.06 | .13 | 120/75 | 4.87 |
| 27 | PCOS | 28 | 3.71 | 3.98 | 2.20 | 3.19 | .13 | 102/63 | 4.61 |
| 28 | PCOS | 31 | .82 | 4.49 | 2.48 | 3.39 | .14 | 125/78 | 5.19 |
| 29 | PCOS | 33 | 12.82 | 18.56 | 10.26 | 14.01 | .59 | 112/64 | 5.50 |
| 30 | PCOS | 29 | 8.99 | 9.76 | 5.40 | 7.37 | .31 | 130/80 | 5.20 |
| 31 | PCOS | 40 | 4.58 | 4.88 | 2.57 | 4.90 | .15 | 104/60 | 3.92 |
| 32 | PCOS | 28 | 11.62 | 8.16 | 2.03 | 5.18 | .24 | 104/79 | 6.09 |
| 33 | PCOS | 30 | 82.49 | 70.31 | 28.02 | 33.63 | .55 | 144/91 | 5.42 |
| 34 | PCOS | 27 | 8.99 | 4.60 | 2.54 | 6.73 | .15 | 112/78 | 5.36 |
| 35 | PCOS | 30 | 29.48 | 6.37 | 3.52 | 4.80 | .20 | 116/72 | 5.51 |
| 36 | PCOS | 31 | 42.02 | 13.94 | 5.61 | 7.65 | .32 | 126/80 | 4.86 |
| 37 | PCOS | 29 | 17.67 | 8.17 | 4.51 | 6.16 | .26 | 133/80 | 5.45 |
| 38 | PCOS | 30 | 10.36 | 3.06 | 1.69 | 2.31 | .10 | 103/56 | 5.11 |
| 39 | PCOS | 25 | 3.45 | 3.94 | 2.18 | 2.97 | .12 | 103/68 | 4.96 |
| 40 | PCOS | 29 | 18.62 | 16.03 | 8.86 | 12.10 | .51 | 134/69 | 5.09 |
| 41 | PCOS | 36 | - | 5.24 | 2.90 | 3.95 | .17 | 112/70 | 5.27 |
| 42 | PCOS | 24 | 1.99 | 3.41 | 1.88 | 2.57 | .11 | 139/91 | 5.98 |
| 43 | PCOS | 29 | 7.49 | 16.53 | 9.14 | 12.47 | .52 | 112/74 | 4.66 |
| 44 | PCOS | 23 | 4.07 | 6.65 | 3.68 | 5.02 | .21 | 120/60 | 5.17 |
| 45 | PCOS | 36 | 59.87 | 11.07 | 5.26 | 7.18 | .30 | 122/79 | 4.82 |
| 46 | PCOS | 24 | 5.55 | 6.20 | 3.43 | 6.85 | .20 | 114/80 | 4.73 |
| 47 | PCOS | 27 | 22.97 | 5.98 | 3.31 | 4.51 | .19 | 128/74 | 4.80 |
| 48 | PCOS | 23 | 5.29 | 8.05 | 4.45 | 6.07 | .25 | 122/78 | 4.88 |
| 49 | PCOS | 32 | 2.37 | 5.20 | 2.88 | 3.93 | .16 | 125/78 | 5.37 |
| 50 | PCOS | 27 | 2.63 | 7.33 | 4.05 | 5.53 | .23 | 125/76 | 5.33 |
| 51 | PCOS | 29 | 13.71 | 7.72 | 4.27 | 5.82 | .24 | 124/82 | 4.69 |
| 52 | PCOS | 30 | 4.46 | 7.45 | 4.12 | 5.62 | .24 | 122/78 | 4.56 |
| 53 | PCOS | 29 | 6.01 | 3.71 | 2.05 | 3.29 | .12 | 109/65 | 4.74 |
| 54 | PCOS | 32 | - | 26.24 | 14.50 | 19.80 | .83 | 110/74 | 5.03 |
| 55 | PCOS | 31 | - | 16.56 | 9.15 | 12.49 | .52 | 120/78 | 5.51 |
| 56 | Control | 33 | - | 5.11 | 2.83 | 3.86 | .16 | 123/73 | 5.48 |
| 57 | Control | 24 | - | 17.36 | 9.59 | 13.10 | .55 | 137/79 | 5.72 |
| 58 | Control | 40 | 5.76 | 3.04 | 2.04 | 4.69 | .08 | 122/70 | 6.01 |
| 59 | Control | 37 | 5.17 | 4.18 | 2.31 | 4.22 | .13 | 104/67 | 5.29 |
| 60 | Control | 24 | 2.25 | 9.49 | 5.25 | 8.47 | .30 | 112/78 | 4.41 |
| 61 | Control | 28 | 25.95 | 6.94 | 3.83 | 5.23 | .22 | 112/75 | 4.40 |
| 62 | Control | 33 | 3.95 | 2.92 | 1.61 | 2.20 | .09 | 120/78 | 5.39 |
| 63 | Control | 39 | - | 11.42 | 6.31 | 8.62 | .36 | 110/63 | 4.90 |
| 64 | Control | 23 | 3.69 | 9.70 | 5.36 | 7.32 | .31 | 92/60 | 4.82 |
| 65 | Control | 28 | 2.58 | 4.13 | 2.25 | 3.07 | .13 | 123/68 | 6.42 |
| 66 | Control | 25 | 12.66 | 7.08 | 3.91 | 5.34 | .22 | 120/74 | 5.20 |
| 67 | Control | 31 | 5.21 | 4.30 | 2.07 | 3.82 | .04 | 127/71 | 5.72 |
| 68 | Control | 34 | 10.86 | 8.66 | 4.79 | 6.63 | .27 | 129/76 | 5.90 |
| 69 | Control | 34 | 9.72 | 19.65 | 10.86 | 14.82 | .62 | 121/78 | 5.33 |
| 70 | Control | 36 | 1.99 | 5.01 | 2.77 | 3.78 | .16 | 105/65 | 5.15 |
| 71 | Control | 31 | 4.50 | 3.82 | 2.03 | 3.90 | .12 | 127/68 | 5.01 |
| 72 | Control | 29 | 14.30 | 7.96 | 4.40 | 6.42 | .25 | 112/69 | 4.97 |
| 73 | Control | 32 | 9.16 | 6.84 | 3.78 | 5.16 | .22 | 110/61 | 4.79 |
| 74 | Control | 31 | 8.63 | 6.13 | 3.39 | 4.62 | .19 | 110/74 | 4.80 |
| 75 | Control | 35 | 6.02 | 8.65 | 4.96 | 6.53 | .27 | 112/71 | 5.52 |
| 76 | Control | 27 | 6.06 | 3.85 | 2.13 | 2.91 | .12 | 112/78 | 4.84 |
| 77 | Control | 25 | 2.82 | 8.05 | 4.45 | 7.96 | .25 | 106/63 | 4.62 |
| 78 | Control | 38 | 6.61 | 21.64 | 11.96 | 16.33 | .68 | 119/75 | 5.34 |
| 79 | Control | 26 | 8.41 | 3.83 | 1.72 | 4.73 | .10 | 130/80 | 6.02 |
| 80 | Control | 33 | 11.49 | 15.82 | 8.74 | 11.93 | .50 | 112/74 | 4.60 |
| 81 | Control | 31 | 8.74 | 7.45 | 4.12 | 5.62 | .24 | 110/70 | 5.80 |
| 82 | Control | 27 | 8.27 | 7.69 | 4.25 | 5.80 | .24 | 124/80 | 5.31 |
| 83 | Control | 25 | 11.98 | 11.42 | 6.31 | 8.62 | .36 | 106/63 | 4.71 |
| 84 | Control | 26 | 4.65 | 13.01 | 7.19 | 9.82 | .41 | 138/70 | 5.65 |
| 85 | Control | 31 | 6.53 | 1.44 | .80 | 1.43 | .05 | 117/88 | 5.17 |
| 86 | Control | 30 | 7.11 | 4.69 | 2.59 | 3.54 | .15 | 106/82 | 5.05 |
| 87 | Control | 28 | 4.67 | 4.23 | 2.34 | 3.19 | .13 | 102/60 | 4.94 |
| 88 | Control | 27 | 7.45 | 9.73 | 5.37 | 7.34 | .31 | 127/77 | 4.93 |
| 89 | Control | 32 | 9.04 | 9.17 | 5.07 | 6.92 | .29 | 116/73 | 4.98 |
| 90 | Control | 33 | 8.63 | 6.38 | 3.53 | 4.82 | .20 | 110/74 | 5.78 |
| 91 | Control | 39 | 6.38 | 3.49 | 1.93 | 2.64 | .11 | 100/58 | 4.22 |
| 92 | Control | 33 | 6.43 | 1.94 | 1.07 | 3.35 | .06 | 118/78 | 5.20 |
| 93 | Control | 26 | 9.52 | 15.56 | 8.60 | 11.74 | .49 | 124/81 | 5.70 |
| 94 | Control | 27 | 2.06 | 6.71 | 3.71 | 5.06 | .21 | 110/60 | 4.30 |
| 95 | Control | 27 | 2.22 | 2.85 | 1.58 | 2.78 | .09 | 103/68 | 4.76 |
| 96 | Control | 29 | 4.02 | 7.34 | 3.63 | 4.96 | .21 | 98/60 | 4.73 |
| 97 | Control | 32 | 3.46 | 4.42 | 2.44 | 3.33 | .14 | 114/77 | 5.24 |
| 98 | Control | 30 | 6.38 | 34.65 | 19.15 | 26.14 | 1.09 | 122/67 | 4.90 |
| 99 | Control | 26 | 8.79 | 5.09 | 2.81 | 5.43 | .16 | 129/79 | 4.72 |
| 100 | Control | 28 | 14.08 | 2.24 | 1.24 | 2.28 | .07 | 138/83 | 5.05 |
| 101 | Control | 35 | 5.10 | 23.77 | 13.13 | 17.93 | .75 | 110/74 | 4.90 |
| 102 | Control | 34 | 38.90 | 19.62 | 10.84 | 14.80 | .62 | 123/70 | 4.66 |
| 103 | Control | 28 | 4.52 | 6.76 | 3.74 | 5.10 | .21 | 118/70 | 4.99 |
| 104 | Control | 31 | 9.05 | 11.21 | 5.46 | 9.08 | .21 | 104/70 | 4.86 |
| 105 | Control | 31 | 2.92 | 17.03 | 9.41 | 12.85 | .54 | 138/78 | 4.76 |
| 106 | Control | 31 | 3.20 | 3.70 | 2.04 | 2.79 | .12 | 110/74 | 5.27 |
| 107 | Control | 32 | 5.94 | 19.27 | 10.65 | 14.54 | .61 | 124/80 | 5.50 |
| 108 | Control | 39 | 10.13 | 5.95 | 3.29 | 4.49 | .19 | 109/64 | 4.62 |
| 109 | Control | 26 | 2.05 | 4.26 | 2.35 | 3.21 | .13 | 100/60 | 4.58 |
| 110 | Control | 37 | 6.21 | 36.10 | 19.95 | 27.23 | 1.14 | 120/75 | 4.79 |
| 111 | Control | 27 | 8.24 | 2.39 | 1.22 | 2.31 | .07 | 107/63 | 5.31 |
| 112 | Control | 36 | 17.55 | 8.73 | 5.22 | 5.61 | .13 | 104/64 | 4.74 |
| 113 | Control | 31 | 10.90 | 11.30 | 6.24 | 8.52 | .36 | 127/75 | 4.86 |
| 114 | Control | 33 | 8.67 | 3.63 | 2.01 | 2.74 | .11 | 125/84 | 4.80 |
| 115 | Control | 25 | - | 5.51 | 3.04 | 4.15 | .17 | 100/60 | 4.40 |
| 116 | Control | 27 | 7.09 | 3.04 | 1.63 | 3.55 | .09 | 120/74 | 5.60 |
| 117 | Control | 38 | 7.08 | 4.25 | 2.35 | 4.03 | .13 | 105/65 | 4.91 |
| 118 | Control | 28 | 9.17 | 12.02 | 6.64 | 9.17 | .38 | 114/61 | 4.84 |
| 119 | Control | 34 | - | 19.27 | 10.65 | 14.54 | .61 | 109/60 | 5.41 |
| 120 | Control | 25 | 34.26 | 12.92 | 7.14 | 9.75 | .41 | 117/78 | 4.74 |
| 121 | Control | 28 | - | 23.01 | 12.72 | 17.36 | .73 | 102/78 | 5.09 |
| 122 | Control | 26 | 31.75 | 20.20 | 11.16 | 15.24 | .64 | 100/66 | 5.16 |
| 123 | Control | 27 | 1.36 | 4.89 | 2.70 | 3.69 | .15 | 110/61 | 4.66 |
| 124 | Control | 30 | 1.20 | 3.17 | 1.75 | 2.39 | .10 | 109/68 | 4.30 |

UACR, the urinary albumin to creatinine ratio; KapU, human immunoglobulin/light chain κ-type; LamU, human immunoglobulin/light chain λ-type; α1-MU, α1-Microglobulin; β2-MU, β2-Microglobulin; GLU, fasting glucose.

**Supplementary Table 3. Parameter values in isolated human granulosa cells**

| **No.** | **Group** | **Age** | **Serum T** **(**ng/mL**)** | **T in FF** **(**ng/mL**)** |
| --- | --- | --- | --- | --- |
| 1 | Control | 28 | 0.11 | 9.12 |
| 2 | Control | 30 | 0.12 | 6.10 |
| 3 | Control | 27 | 0.171 | 3.02 |
| 4 | Control | 36 | 0.198 | 6.57 |
| 5 | Control | 26 | 0.21 | 5.02 |
| 6 | PCOS+normal T | 28 | 0.22 | 9.88 |
| 7 | PCOS+normal T | 30 | 0.32 | 6.49 |
| 8 | PCOS+normal T | 27 | 0.36 | 7.00 |
| 9 | PCOS+normal T | 36 | 0.30 | 8.72 |
| 10 | PCOS+normal T | 26 | 0.21 | 3.57 |
| 11 | PCOS+high T | 29 | 0.55 | 13.82 |
| 12 | PCOS+high T | 30 | 0.724 | 12.58 |
| 13 | PCOS+high T | 27 | 0.79 | 19.00 |
| 14 | PCOS+high T | 36 | 0.49 | 14.02 |
| 15 | PCOS+high T | 26 | 0.51 | 18.00 |

T, testosterone; FF, follicular fluid.
